# From English to Chinese, Japanese, and Russian: extending research visibility with language translations of a conference slide presentation

**DOI:** 10.5195/jmla.2017.106

**Published:** 2017-01

**Authors:** Lilian Hoffecker, Dana Abbey

## Abstract

**Objective:**

The research demonstrates that a conference slide presentation translated into non-English languages reaches significantly larger and different audiences than an English presentation alone.

**Methods:**

The slides of a presentation from the Medical Library Association annual meeting were translated from English to Chinese, Japanese, and Russian and posted along with the English version to SlideShare, an open slide-hosting website. View counts, traffic sources, and geographic origins of the traffic for each language version were tracked over a twenty-two-month period.

**Results:**

Total view counts for all 4 language versions amounted to 3,357 views, with the Chinese version accounting for 71% of the total views. The trends in view counts over time for the Japanese, Russian, and English versions were similar, with high interest at the beginning and a rapid drop and low level of viewing activity thereafter. The pattern of view counts for the Chinese version departed considerably from the other language versions, with very low activity at the beginning but a sharp rise 10 months later. This increase in activity was related to access to the presentations via a Taiwanese website that embedded the SlideShare website code.

**Conclusions:**

Language translation can be a difficult and time-consuming task. However, translation of a conference slide presentation with limited text is an achievable activity and engages an international audience for information that is often not noticed or lost. Although English is by far the primary language of science and other disciplines, it is not necessarily the first or preferred language of global researchers. By offering appropriate language versions, the authors of presentations can expand the reach of their work.

## INTRODUCTION

Researchers are highly interested in making their work visible through a variety of venues, including contributions at conferences and formal publications. Paper or poster presentations are common to most professional conferences, allowing researchers to speak to and interact directly with their peers. The advantage of conference presentations, therefore, is their personal and often informal nature, allowing one-on-one conversations, but this also means they reach only the conference members who attend the paper presentation or stop by to view a poster. As a result of the open access movement [1] and the availability of online sharing tools, proactive researchers now have publication options within licensing and copyright agreements to make their work widely and globally accessible.

However, a global audience does not necessarily mean an English-fluent audience. Although English is spoken as a first language by only 5% of the world’s population [2, 3], it has become the lingua franca of science, and many researchers believe that the only way to become an internationally recognized investigator is to communicate in English [4]. Language barriers may be impediments to career advancement [5, 6] and potentially to collaboration [7, 8].

Instead of having non-native English speakers, or non-Anglophones, attempt communication in English, what if English works were translated to other languages? Could non-Anglophones be missing pertinent research information due to a language barrier? Just as translation from other languages to English is challenging, translating from English to other languages is equally, if not more, difficult, depending on the language. While translation of an entire article is a major effort, translating information from bulleted lists on a slide presentation or an article abstract may be a feasible undertaking.

To test the hypothesis that non-English-speaking scholars are not discovering relevant literature due to a language barrier, the authors undertook a small pilot study to examine the visibility of a presentation from the 2014 Medical Library Association annual meeting by translating it into Chinese, Japanese, and Russian. While a conference presentation on library research is not necessarily representative of research presentations in general, we believe our findings are applicable to other fields of scholarship in which English is the dominant language. Chinese, Japanese, and Russian were selected in part because of their complexity for native speakers of English. The Foreign Services Institute of the US State Department, for example, classifies them as among the most difficult languages to learn for Anglophones. Native Chinese, Japanese, or Russian speakers would, therefore, presumably find it more challenging to publish in English compared with native speakers of Romance or other languages that are linguistically closer to English [9, 10]. Here, we present our findings and their implications for making research visible worldwide.

## METHODS

In May 2014, we presented a paper in Chicago, Illinois, at the Medical Library Association annual meeting. The presentation, “What’s the Difference Between Altmetrics and Other Measures of Research Influence? Exploring Alternative Metrics, Impact Factors, and More,” was fifteen minutes in length with a total of seventeen slides, attracting roughly fifty meeting attendees. It explained different bibliometric methods (altmetrics, citation rates, h-index, and others) and compared two commercial services, Altmetric.com [11] and PlumX [12], which gather social media and other non-citation data to assess research visibility. PlumX has since become a subsidiary of EBSCO Information Services [13].

Prior to the meeting, we had the slides translated into Chinese, Japanese, and Russian, although the live presentation at the meeting was based only on the English version. One of the authors (Hoffecker) translated the presentation into Japanese, while two volunteers with a familiarity of Russian and Chinese translated the presentation into these languages. None were professionally trained translators.

In June 2014, all language versions, including English, were posted to SlideShare [14], an open slide-hosting website. While there are other resources for sharing slide presentations (e.g., FigShare), SlideShare provides useful analytics and has potential for a large audience with over seventy million visitors [15]. Each slide of a non-English version was alternated with the corresponding English slide, thus doubling the total number of slides. Over a twenty-month period, starting June 11, 2014, until April 11, 2016, we tracked data on the number of views, the source of traffic, and the geographic location of the views. Visitors to the slide presentations were not targeted or solicited.

## RESULTS

**Figure 1:**
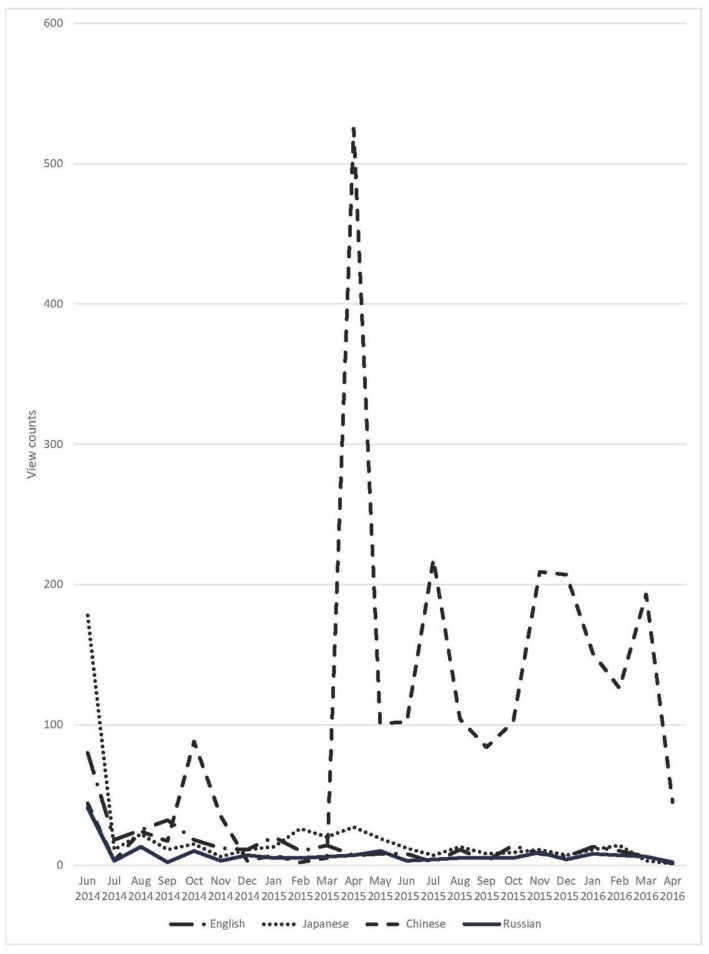
View counts over time for each language version

During the 22-month period, the 4 versions accumulated a total of 3,357 view counts. The Chinese version accumulated the majority of views (2,395 views, 71.3% of view counts), followed by the Japanese (455, 13.6%), English (337, 10.0%), and Russian (170, 5.1%) versions. Over time, the pattern of views shifted appreciably as shown in Figure 1. The English, Japanese, and Russian versions followed parallel patterns displaying high view counts at the beginning followed by a sharp drop. The trendline of the Chinese version, however, departed considerably from those of the other languages, showing very little activity at the beginning and rising dramatically in April 2015. The activity then plummeted but remained higher than the activity before April 2015.

Slideshare.net provides data on how viewers reach the slides by identifying five categories of traffic sources [16]: “direct” (clicking a link in an email or typing the uniform resource locator [URL] into a browser), “referral” (clicking a link in another web page), “search” (clicking a link displayed in the results from a search engine), “slideshare” (clicking a link within SlideShare), “embed” (clicking a link from another web page, similar to “referral,” but one that uses Slideshare’s embed-code), and “social” (clicking a link in social media sites such as Facebook or Twitter). Most viewers of the Japanese, Russian, and English versions arrived at the slides via the “direct” method, and the “slideshare” method was the second most popular method. Specifically, viewers of the Japanese, English, and Russian language version arrived at the presentation 81%, 76%, and 56% of the time using the “direct” method, respectively. The “slideshare” method was the route taken by viewers of the Japanese, English, and Russian versions 14%, 16%, and 35% of the time, respectively. By contrast, the most common way Chinese viewers reached the slides was via “embed” (56%), while the second most frequent method was “referral” (17%). In fact, one Taiwanese site, Library Views [17], seemed to be responsible for much of this traffic. Table 1 shows the top five countries for the traffic sources for each language version.

**Table 1:** Top five countries for the traffic sources of different language versions (June 11, 2014 to April 7, 2016)

Top countries for Chinese version	View counts	Top countries for Japanese version	View counts	Top countries for Russian version	View counts	Top countries for English version	View counts
Taiwan	1,685	United States	231	United States	138	United States	231
United States	314	Germany	78	Russian Federation	14	Germany	22
Hong Kong	118	France	40	Ukraine	12	France	15
China	69	Japan	29	South Korea	2	Russian Federation	9
Anonymous (geographic location was indeterminable)	35	Canada	14	France	2	Japan	9

## DISCUSSION

While a view of an uploaded file is not comparable to attendance at a live presentation, total views can give an estimate of the visibility of a presentation. In fact, the total view count (3,557) of our presentation indicates that the slides were noticed by hundreds or even thousands of individuals. The English version alone accounted for 334 views, but the Chinese and Japanese versions attracted greater attention, indicating that translation to different languages can play a significant role in raising the profile of a conference presentation. Compared to an estimated audience of 50 individuals at the live meeting, the audience that the slides in their various language versions reached was not only a worldwide audience, but also a global native audience.

Furthermore, compared with a full article, translating a slide presentation is not as onerous and time-consuming, and even if a professional translation service is involved, not as costly. Conference presentations are often lost since they are not published like articles, and even the abstracts may or may not be published in an issue of a society’s journal. For instance, the Medical Library Association does not reproduce the presentation abstracts in an issue of the *Journal of the Medical Library Association* but instead gives access only to registered meeting participants.

How viewers get to the slides is an important consideration. The “direct” method may be a common way to reach presentations because users often share links via email. However, the Chinese version benefitted considerably by being embedded or linked in a website (considered an “embed” or “referral”). Its rise in views about ten months after upload is related to its discovery by a website in Taiwan and not due to interest in China. While the low activity from China may be related to censorship actions taken by its government to block SlideShare starting in 2012 [18], it may also be related to the fact that SlideShare is essentially an English-language website. This characteristic may also explain why Japan is fourth on the list of countries accessing the Japanese version (Table 1). Different than SlideShare, sites like Amazon or Google have unique versions of these sites for major languages (e.g., Amazon.co.jp or Google.co.jp are Japanese versions of these sites), making it easier for non-English shoppers and searchers to navigate them.

A few qualifications regarding this study should be considered. First, we relied on volunteers for the translations. For more accurate translations, a professional service is necessary though costly. Second, although data analytics provided by SlideShare are useful, they must be interpreted as overall trends rather than precise measures. For example, if view counts were monitored over six months instead of twenty-two months, we would have concluded that there was not much demand for a Chinese language version. Finally, the topic of the slides was specialized to librarians with an interest in bibliometrics. If the selected topic was more general or trendy, our language choices and results might have been quite different.

We see opportunities for librarian involvement in assisting researchers with raising the visibility of their scholarly work through language translation. Librarians can help identify appropriate open access venues for posting translated items, select metatags prior to translation, and match appropriate languages for translation to the particular research topic. Librarians can also work with researchers to continually “feed” social media (e.g., Twitter, Facebook, blogs, websites) and professional networking sites (e.g., LinkedIn, ResearchGate) with the translated works. Just as journal articles decline in interest and usage after their initial publication, these venues need continual feeds of information to stay in readers’ awareness.
